# Molecular profiles of red blood cells across geographic, pathological, and age-related perspectives: translational insights

**DOI:** 10.3389/fmed.2026.1798592

**Published:** 2026-04-30

**Authors:** Khawla Yousif Almadhaani, Alaa Muayad Altaie, Rifat Hamoudi

**Affiliations:** 1 Research Institute for Medical and Health Sciences, University of Sharjah, Sharjah, United Arab Emirates; 2 Department of Clinical Sciences, College of Medicine, University of Sharjah, Sharjah, United Arab Emirates; 3 Blood Transfusion and Research Center, Emirates Health Services, Sharjah, United Arab Emirates; 4 Center of Excellence for Precision Medicine, Research Institute of Medical and Health Sciences, University of Sharjah, Sharjah, United Arab Emirates; 5 BIMAI-Lab, Biomedically Informed Artificial Intelligence Laboratory, University of Sharjah, Sharjah, United Arab Emirates; 6 ASPIRE Precision Medicine Research Institute Abu Dhabi, University of Sharjah, Sharjah, United Arab Emirates; 7 Division of Surgery and Interventional Science, University College London, London, United Kingdom

**Keywords:** CD36, CD47, ICAM-4, PIEZO1, RBC aging, red blood cell

## Abstract

Red blood cells (RBCs), formerly viewed as mere oxygen transporters, are now acknowledged as dynamic biological entities with intricate molecular characteristics which impact their deformability, lifespan, and interactions within the vascular system. Progress in molecular hematology has elucidated how modifications in RBC proteins, lipids, ion channels, and adhesion molecules influence physiological adaptability, pathogenic mechanisms, and transfusion results. Inherited and acquired illnesses, such as sickle cell disease, thalassemia, malaria, human immunodeficiency virus (HIV), and metabolic disorders, highlight how alterations in membrane structure, phospholipid asymmetry, and signaling pathways exacerbate hemolysis, restrict circulation, and provoke inflammation or thrombosis. Geographic and environmental pressures, such as hypoxia at elevated altitudes, emphasize the variety of molecular methods that facilitate oxygen delivery. The integration of these insights into clinical practice reveals that molecular markers such as Band 3, PIEZO1, ICAM-4, CD36, and CD47 are becoming significant diagnostic and prognostic instruments, while targeted therapies focusing on ion channels, oxidative pathways, and adhesion molecules are creating new therapeutic possibilities. These results highlight that RBC molecular profiling is enhancing our comprehension of erythrocyte biology while also transforming diagnostics, transfusion medicine, and customized therapy in hematology.

## Introduction

Red blood cells (RBCs) are extensively researched cell types in human biology, however their molecular intricacy continues to uncover novel insights with direct therapeutic significance. Beyond their primary role in oxygen transport, RBCs exhibit complex structural and biochemical adaptations that maintain their deformability, ensure survival throughout their 120-day lifespan, and facilitate passage through the microvasculature (1–3). The erythrocyte membrane, composed of proteins, lipids, and glycoproteins arranged in a defined architecture, integrates both signaling and mechanical properties that are essential for vascular homeostasis (4–7).

Although the basic biology of RBCs is well understood, significant information gaps persist concerning how molecular changes in many contexts such as geographic adaptation, hereditary and acquired hematological diseases, infections, and aging intersect to influence clinical outcomes. Environmental stressors such hypoxia at elevated altitudes, genetic conditions such as sickle cell anemia and thalassemia, and viral diseases including malaria and human immunodeficiency virus (HIV) exemplify the several dimensions along which RBCs experience structural and functional alterations (8–11). These alterations often present as modified membrane protein activity, phospholipid asymmetry, or aberrant expression of adhesion molecules, ultimately resulting in compromised deformability, hemolysis, and pathogenic interactions with the vasculature (12–16).

Recent advancements in molecular hematology, proteomics, and imaging technologies have yielded unparalleled clarity into these processes. Mechanistic investigations of Band 3, PIEZO1, and Gardos channel activity have clarified the role of ion flux in volume regulation and hemolysis (17–20). Research on flippases, scramblases, and glycoproteins, including glycophorin A, ICAM-4, CD36, and CD47, has uncovered their dual functions in RBC stability and interactions with the immune system or pathogens (21–25). Despite these advancements, reviews frequently address these subjects in isolation, concentrating on either structural biology or clinical diseases.

This review aims to integrate diverse perspectives into a unified framework. By synthesizing molecular abnormalities observed across geography, pathology, and age, we delineate both common and distinct mechanisms that regulate RBC stability. In addition, we evaluate the translational relevance of these molecular insights in clinical practice, with emphasis on diagnostics, transfusion medicine, and emerging therapeutic strategies. This integrative approach underscores the evolving view of RBCs as dynamic molecular systems, whose study advances understanding in hematology as well as broader systemic health and disease management.

## Molecular composition of RBCs

RBCs are highly specialized to perform the vital task of oxygen transport (1). Their unique structure is adapted to enhance their flexibility and endurance during traveling through the circulatory system (2, 3). This adaptation is due to the RBCs’ membrane composition, which consists of a complex network of membrane proteins, phospholipids, and cytoskeletal elements that work together to ensure functionality (3, 4).

RBCs exhibit a highly specialized and sophisticated structure optimized for their primary role in oxygen transport. The RBC membrane is composed of a delicate balance of lipids, proteins, and a cytoskeletal framework that grants the cell its characteristic biconcave shape and remarkable flexibility (4, 5, 26). This structural adaptation allows RBCs to endure the mechanical stresses of circulating through narrow capillaries while simultaneously maximizing surface area to facilitate efficient gas exchange (1). Major molecular elements like integral and peripheral membrane proteins, along with phospholipids, work synergistically to manage the stability of the cells, regulate ion transport, and secure deformability (1).

**Figure 1:**
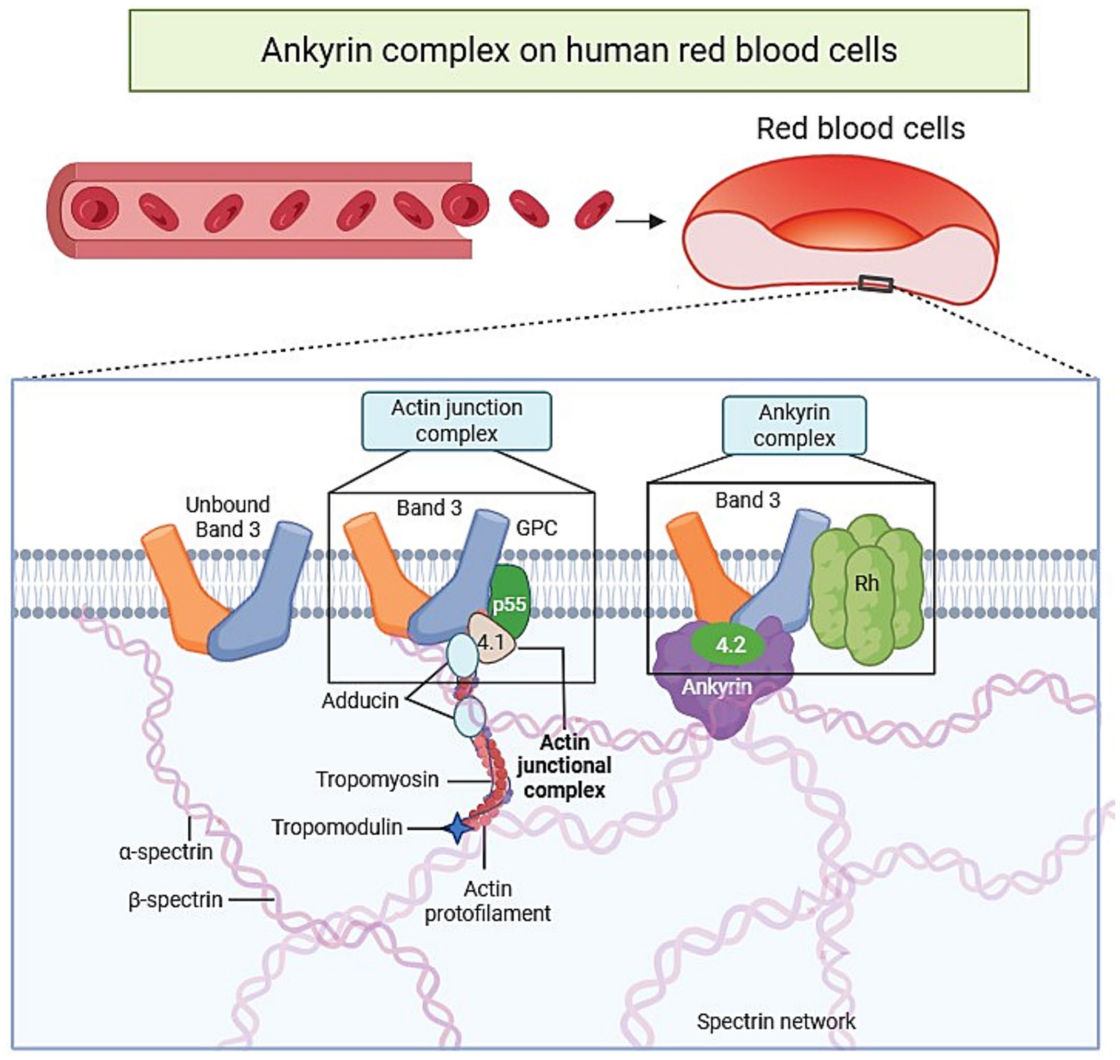
A schematic depiction of the membrane structure of red blood cells (RBCs) highlighting key functional components. The RBC membrane comprises three key structural components: the lipid bilayer, transmembrane proteins, and the supporting cytoskeletal network. Band 3 and glycophorins are notable integral proteins that serve as anchoring sites for the cytoskeleton in addition to mediating ion exchange and surface charge control. The membrane skeleton is anchored to the bilayer via two primary macromolecular assemblies: the ankyrin complex, which involves the interaction of band 3 with protein 4.2 and the Rh complex that connects spectrin filaments, and the actin junctional complex, which includes band 3, glycophorin C (GPC), protein 4.1R, p55, and adducin, establishing an essential point for cytoskeletal cross-linking. Short actin protofilaments are reinforced by tropomyosin and tropomodulin, whereas α- and β-spectrin heterodimers form a two-dimensional lattice beneath the bilayer. These interconnected molecular systems maintain the RBC’s distinctive biconcave shape, facilitate its remarkable deformability, and uphold its mechanical durability during continuous microcirculatory passage. Created with BioRender.com.

RBCs membrane proteins are fundamental in maintaining the cell’s integrity, ensuring flexibility, and facilitating efficient gas exchange. This network includes key proteins such as band 3 (anion exchanger 1), glycophorins (particularly glycophorin A), Rh-associated glycoproteins (RhAG), spectrin, ankyrin, protein 4.1R, protein 4.2, actin, and various ion channels including PIEZO1 and the Gardos channel. Together, these proteins coordinate membrane stability, cytoskeletal architecture, and ionic balance, ensuring the deformability required for efficient passage through the microvasculature (2). Based on their structural organization and localization within the membrane, RBC proteins are broadly classified into two major groups: integral membrane proteins and peripheral membrane proteins. Each plays a distinctive role in the structural and functional dynamics of the RBCs (1, 4, 6). Integral membrane proteins span the lipid bilayer and are essential for ion transport and gas exchange. Among them, band 3 is the most prominent, representing the most abundant integral protein in the RBCs membrane. Band 3 is present in over a million copies per cell and has important functions concerning ion exchange and mechanical support (7, 27). Band 3 exists in two forms: tetrameric and dimeric. The tetrameric form has an interaction with ankyrin that forms a macrocomplex which anchors the membrane to the underlying cytoskeleton, a critical activity for both structural support and functional gas exchange. The dimeric form of the band 3 protein, interacts with adducin to link with the junctional complex that connects the cytoskeleton to the membrane. In addition to band 3, other key intrinsic proteins include glycophorin A (GPA), which contributes to the negative surface charge of the RBCs membrane and thereby prevents cell aggregation, and Rh-associated glycoproteins (RhAG), which participate in ion transport to maintain proper RBC hydration (11) (Figure 1). In addition to its structural role, band 3 functions as a central regulator of red blood cell metabolism and homeostasis through its interactions with glycolytic enzymes and cytoskeletal proteins, highlighting its critical role in RBC lifespan and functional integrity (27).

In contrast, peripheral membrane proteins are anchored to the cytoplasmic surface of the membrane and are essential for maintaining the characteristic shape and mechanical properties of RBCs. The most prominent of these is spectrin, a filamentous protein that forms a supportive lattice beneath the membrane, critical for the biconcave disk shape and for providing the flexibility needed to traverse narrow capillaries (9). Ankyrin serves as a linker between spectrin and band 3, thereby stabilizing the interaction between the cytoskeleton and the lipid bilayer (8). Additionally, protein 4.1R connects spectrin to F-actin at junctional complexes, a key process in preserving cytoskeletal homeostasis (9). Collectively, these proteins enable RBCs to withstand mechanical stress while preserving structural integrity and ensuring efficient physiological function during circulation (Figure 1).

## Molecular mechanisms underlying RBC alteration

### ATP-dependent regulation of RBC function

Adenosine triphosphate (ATP) is essential for preserving the structural integrity and functional equilibrium of red blood cells. Although lacking mitochondria, red blood cells depend on glycolysis for their principal energy supply, and sufficient ATP concentrations are crucial for maintaining membrane flexibility and cytoskeletal structure. ATP directly facilitates the stability of membrane cytoskeleton connections by modulating spectrin actin dynamics and preserving the attachment of integral proteins, such as band 3, to the cytoskeleton (28).

Moreover, ATP is essential for ion homeostasis via the function of membrane transporters, specifically the Na^+^/K^+^-ATPase and Ca^2+^-ATPase pumps, which sustain intracellular ionic equilibrium and prevent calcium excess. Disruption of ATP availability causes deprived ion transport, leading to elevated intracellular Ca^2+^ levels, activation of the Gardos channel, cellular dehydration, and diminished deformability (29, 30). ATP depletion correlates with increased membrane stiffness, increased vesiculation, and premature externalization of phosphatidylserine, all of which facilitate rapid erythrocyte senescence and clearance (21). Moreover, diminished ATP levels intensify oxidative stress-related damage and facilitate cytoskeletal instability, thereby compromising RBC survival. ATP-dependent activities are crucial for preserving erythrocyte deformability, membrane integrity, and lifetime, underscoring their significant involvement in both normal and pathological states (15, 31).

### Membrane proteins and ion channels

Apart from their structural function in preserving the RBC membrane, certain proteins and ion channels coordinate dynamic processes that govern hydration, ionic equilibrium, and cytoskeletal connections. These molecular elements work as active controllers of cellular operations rather than passive structural components, ensuring that RBCs maintain deformability and resilience under persistent mechanical and metabolic stress (15, 19, 21, 32). Disruption of these regulatory pathways via genetic mutations, oxidative damage, or metabolic dysregulation induces maladaptive alterations in volume regulation, membrane integrity, and ion equilibrium, ultimately hastening senescence and propelling the pathophysiology of hemolytic and vaso-occlusive conditions (25, 32, 33).

Band 3, PIEZO1, and the Gardos channel illustrate the essential function of membrane proteins in this environment. Band 3, is pivotal for chloride-bicarbonate exchange and the regulation of intracellular pH. The cytoplasmic domain provides an anchoring hub by connecting the membrane to the spectrin-actin cytoskeleton via ankyrin, so guaranteeing deformability and durability under shear stress. Under oxidative stress, Band 3 experiences conformational clustering that impairs ion transport and destabilizes cytoskeletal interactions. These oxidized clusters serve as senescence indicators, directing RBCs for macrophage-mediated elimination and leading to hemolytic disorders (34, 35). Genetic abnormalities in Band 3, as observed in hereditary spherocytosis and Southeast Asian ovalocytosis, result in morphologically abnormal cells characterized by reduced flexibility and shortened life span due to increased splenic clearance (1). PIEZO1, a mechanosensitive non-selective cation channel, is essential for the regulation of RBC volume in response to mechanical stresses in circulation. In standard physiology, PIEZO1 inhibits osmotic swelling and lysis by regulating Ca^2+^ and Na^+^ fluxes. Gain-of-function mutations disrupt this equilibrium by enhancing cation leakage and facilitating the dehydration of RBCs, resulting in hereditary xerocytosis. Consequently, dehydrated and non-deformable cells exhibit a high susceptibility to hemolysis, with clinical symptoms varying from compensated anemia to transfusion dependency (1, 17, 35). Notably, PIEZO1 regulates RBC activity during blood storage, with subclinical channel activation leading to ion imbalance and microvesiculation. The Gardos channel (KCa3.1), a calcium-activated potassium channel, is essential for regulating volume homeostasis by facilitating potassium efflux and corresponding water loss (1, 7). Under physiological settings, this functions as a protective mechanism against osmotic stress. Excessive activation, as seen in sickle cell disease, leads to pathological dehydration and increased membrane stiffness. This intensifies hemoglobin S polymerization, increases hemolysis, and precipitates 6 events (17). In addition to hereditary hemoglobinopathies, aberrant Gardos channel activity has been associated with storage lesions, wherein increased intracellular Ca^2+^ concentrations progressively activate the channel, leading to cellular shrinkage and diminished deformability under extended refrigeration (11, 36). Collectively, these molecular findings highlight that RBC membrane proteins and ion channels function not only as structural components but also as dynamic regulators of erythrocyte viability. Their dysregulation whether by mutation, oxidative modification, or storage-related stress, induces a series of pathogenic outcomes. Understanding these pathways establishes a basis for therapeutic interventions, such as targeted ion channel inhibitors, antioxidant treatments, and enhanced storage solutions designed to prolong the lifespan and quality of transfused RBCs (11, 36, 37).

### Dynamics of the proteome and remodeling of extracellular vesicles in red blood cells

Red blood cells demonstrate dynamic proteome remodeling despite the absence of nuclei and protein synthesis capabilities, principally by protein modification, selective destruction, and the shedding of extracellular vesicles (EVs) (38, 39). Vesiculation is a crucial mechanism in red blood cell homeostasis, facilitating the elimination of damaged or oxidized membrane proteins, such as aggregated band 3 and denatured hemoglobin complexes, thus maintaining membrane integrity during circulation (38, 40). Excessive vesicle creation leads to membrane loss, reduced surface area, and heightened cellular stiffness (38).

Moreover, red blood cells experience incremental proteome modifications throughout aging and pathological stress, encompassing phosphorylation, oxidation, and structural changes in cytoskeletal and membrane-associated proteins (39, 40). Recent research indicates that red blood cells can assimilate plasma-derived proteins, hence enhancing functional heterogeneity (39). These mechanisms jointly affect membrane stability, deformability, and vulnerability to clearing. From a transfusion standpoint, analogous pathways govern storage-related vesiculation and proteomic alterations, which correlate with diminished post-transfusion recovery and functional performance (38, 39).

### Phospholipids and membrane asymmetry

The asymmetric distribution of phospholipids in the RBC membrane constitutes a dynamic regulatory mechanism crucial for preserving cellular stability, deformability, and viability (1, 41, 42). This asymmetry can be explained by the localization of phosphatidylcholine (PC) and sphingomyelin (SM) to the outer leaflet, while phosphatidylethanolamine (PE) and phosphatidylserine (PS) are mostly confined to the inner leaflet. This polarized arrangement is dynamic, actively sustained by certain lipid transporters, and its disruption acts as both an indicator and facilitator of RBC malfunction (42, 43). The loss of this asymmetry, especially by the exposure of phosphatidylserine on the outer leaflet, has significant physiological consequences, including impaired deformability, increased hemolysis, and the activation of coagulation and immunological recognition (24, 41).

The phospholipid composition directly affects the deformability of RBCs. An unsaturated fatty acid-rich membrane imparts flexibility and fluidity, allowing RBCs to withstand shear forces and cross tiny capillaries. Conversely, the depletion of unsaturated fatty acids or the enrichment of cholesterol diminishes fluidity, resulting in the stiffening of the bilayer and increasing the susceptibility of cells to hemolysis under stress (24, 44). The externalization of phosphatidylserine from the inner to the outer leaflet serves as a molecular “eat-me” signal, identifying aged or damaged RBCs for clearance by macrophages. In transfusion medicine, elevated phosphatidylserine exposure during storage is acknowledged as a characteristic of storage lesion, associated with vesiculation, diminished deformability, and decreased post-transfusion longevity (24, 43).

The asymmetry RBC membrane phospholipids is meticulously controlled by two antagonistic enzymatic systems (1, 41). Flippases, namely P4-ATPases like ATP11A and ATP11C, actively translocate aminophospholipids (PS and PE) from the outer to the inner leaflet by an ATP-dependent mechanism (42, 45). This internal translocation inhibits incorrect phosphatidylserine exposure, hence reducing RBC adhesion to the endothelium or phagocytes under physiological settings (42, 43). Flippase malfunction, either from oxidative stress, caspase cleavage, or genetic lack, disturbs asymmetry and fosters hemolytic abnormalities. ATP11C mutations hinder B-cell growth; yet, in RBCs, diminished flippase activity results in aberrant phosphatidylserine exposure and premature clearance (36, 42). Conversely, scramblases disrupt lipid asymmetry by bidirectionally reallocating phospholipids between leaflets (41, 42, 46). The two principal families are the Ca^2+^-dependent TMEM16 proteins particularly TMEM16F and the caspase-activated Xkr family especially Xkr8. TMEM16F-mediated scrambling is swiftly initiated by increased intracellular calcium levels, as shown during platelet activation or sickle cell crisis, resulting in abrupt phosphatidylserine exposure providing a procoagulant surface (46). Mutations in TMEM16F are responsible for Scott syndrome, a rare coagulopathy marked by deficient phosphatidylserine exposure in platelets (47, 48). In RBCs, abnormal activation of TMEM16F facilitates phosphatidylserine exposure during oxidative stress and promotes hemolysis (36, 48). Xkr8, conversely, is irreversibly activated through caspase cleavage during apoptosis, facilitating persistent PS exposure and assuring identification by phagocytes (49). This process is pertinent to the clearance of senescent neutrophils and may likewise regulate the disposal of terminal RBCs (49).

The pathogenic consequences of altered phospholipid asymmetry are seen in hemoglobinopathies and membranopathies. In sickle cell disease and thalassemia, sustained Ca^2+^ influx activates scramblases and inhibits flippases, resulting in chronic phosphatidylserine exposure, procoagulant activity, and increased RBC clearance (24, 45). In hereditary spherocytosis, structural abnormalities intensify oxidative damage and disrupt lipid structure, accelerating splenic elimination (50). These mechanisms connect lipid biology to thrombosis and inflammation, as externalized phosphatidylserine offers a catalytic surface for coagulation complexes and stimulates immunological responses (48, 50).

The management of phospholipid asymmetry is not only a structural necessity but also a crucial factor influencing RBC longevity, rheology, and hemostatic equilibrium (1, 15, 45). The interaction between flippases and scramblases guarantees dynamic reactivity to physiological signals while limiting the improper exposure of phosphatidylserine under resting conditions (24, 42, 46). The disruption of this delicate equilibrium via oxidative stress, enzymatic inactivation, or genetic mutation induces premature senescence and hemolysis in RBCs (7, 21, 36). Consequently, focusing on these molecular regulators presents a great opportunity for innovative therapeutic techniques designed to maintain RBC deformability, reduce hemolysis, and enhance transfusion results (23, 45).

### Glycoproteins and adhesion molecules

RBC membrane contains a variety of glycoproteins and adhesion molecules crucial for structural integrity, circulation regulation, immunological evasion, and intercellular communication (1, 2, 4). These molecules jointly ensure that RBCs can pass through the vasculature without undesired aggregation, while concurrently participating in physiological interactions that facilitate hemostasis and immune surveillance (2, 15, 51). Dysregulation of their expression or function modifies RBC behavior, hastening senescence and facilitating vascular disease (15, 21, 52).

Glycophorins, especially glycophorin A, are the predominant glycoproteins on the surface of RBCs (1, 4). Their elevated sialic acid concentration provides a significant negative charge to the membrane, hence diminishing nonspecific adherence and aggregation (1, 2, 4). This electrostatic repulsion is essential for maintaining blood fluidity under high-shear situations (2, 15). In addition to their mechanical function, glycophorins act as receptors for pathogens like *Plasmodium falciparum*, facilitating the entry of malaria parasites into RBCs (31, 51). Mutations or modified expression of glycophorins result in two consequences, diminished anti-adhesive function and heightened vulnerability to infection (1, 15, 31).

Intercellular adhesion molecule-4 (ICAM-4) is an erythroid-specific adhesion molecule that engages with several integrins (1, 2, 51). Under typical circumstances, these connections remain dormant; however, under pathological conditions like sickle cell disease, the expression and accessibility of ICAM-4 are augmented, facilitating the adherence of RBCs to endothelial cells and platelets (11, 51). Recent research indicates that ICAM-4 can directly bind to the platelet fibrinogen receptor αIIbβ3 in its high-affinity activated state, establishing ICAM-4 as the first RBC ligand for activated platelets (51). This interaction necessitates both Ig-like domains of ICAM-4 and includes a QXXDV motif within domain D1 that resembles established fibrinogen-binding sequences (51). ICAM-4 also facilitates vaso-occlusive crises in sickle cell disease and promotes thrombus development in wider cardiovascular scenarios (11, 51, 52).

CD36, a multifunctional scavenger receptor, serves a dual function in RBC physiology. It aids in the removal of oxidized lipids and senescent RBCs, acting as a protective mechanism against the accumulation of damaged cells (1, 15, 51). Conversely, CD36 is utilized by pathogens like *Plasmodium falciparum*, facilitating the sequestration of infected RBCs within the microvasculature (31, 51). This promotes parasite survival while impairing circulation, exacerbating severe malaria pathology (31, 51). Elevated CD36 expression or activity signifies a pathogenic enhancement of normally homeostatic clearance mechanisms (1, 15, 31).

CD47, sometimes referred to as the “do not eat me” signal, operates by binding to signal regulatory protein α (SIRPα) on macrophages, thereby preventing phagocytosis (1, 2). Elevated CD47 expression on healthy RBCs promotes immunological tolerance and extends circulation duration, whereas reduced CD47 density on elderly or injured RBCs acts as a clearance signal (15, 21, 22). Variations in CD47 expression carry significant clinical implications (25). In autoimmune hemolytic anemia, reduced CD47 expression promotes red blood cell destruction, whereas in cancer, elevated CD47 levels enable tumor cells to evade immune surveillance (48). Moreover, discrepancies in CD47 signaling during transfusion therapy can influence the survival of transfused red blood cells and modulate alloimmune responses (1, 15, 25).

Glycoproteins and adhesion molecules serve as essential regulators of RBC stability, maintaining erythrocyte deformability, immunological compatibility, and circulatory efficiency (1, 2, 4). Their intricate equilibrium between sticky capacity and anti-adhesive defense influences both the survival of RBCs in circulation and their role in vascular homeostasis (2, 15, 51). The disruption of this equilibrium highlights the pivotal function of glycoproteins in both normal erythrocyte physiology and the development of hemolytic and vaso-occlusive diseases (11, 15, 21, 52).

### Mechanisms of RBC maturation and senescence

RBCs life cycle is defined by strictly regulated processes of maturation and senescence, ensuring that RBCs efficiently perform their oxygen transport function throughout their lifespan. These processes, from erythropoiesis in the bone marrow to eventual clearance from circulation, are controlled by intricate molecular mechanisms (10).

**Figure 2:**
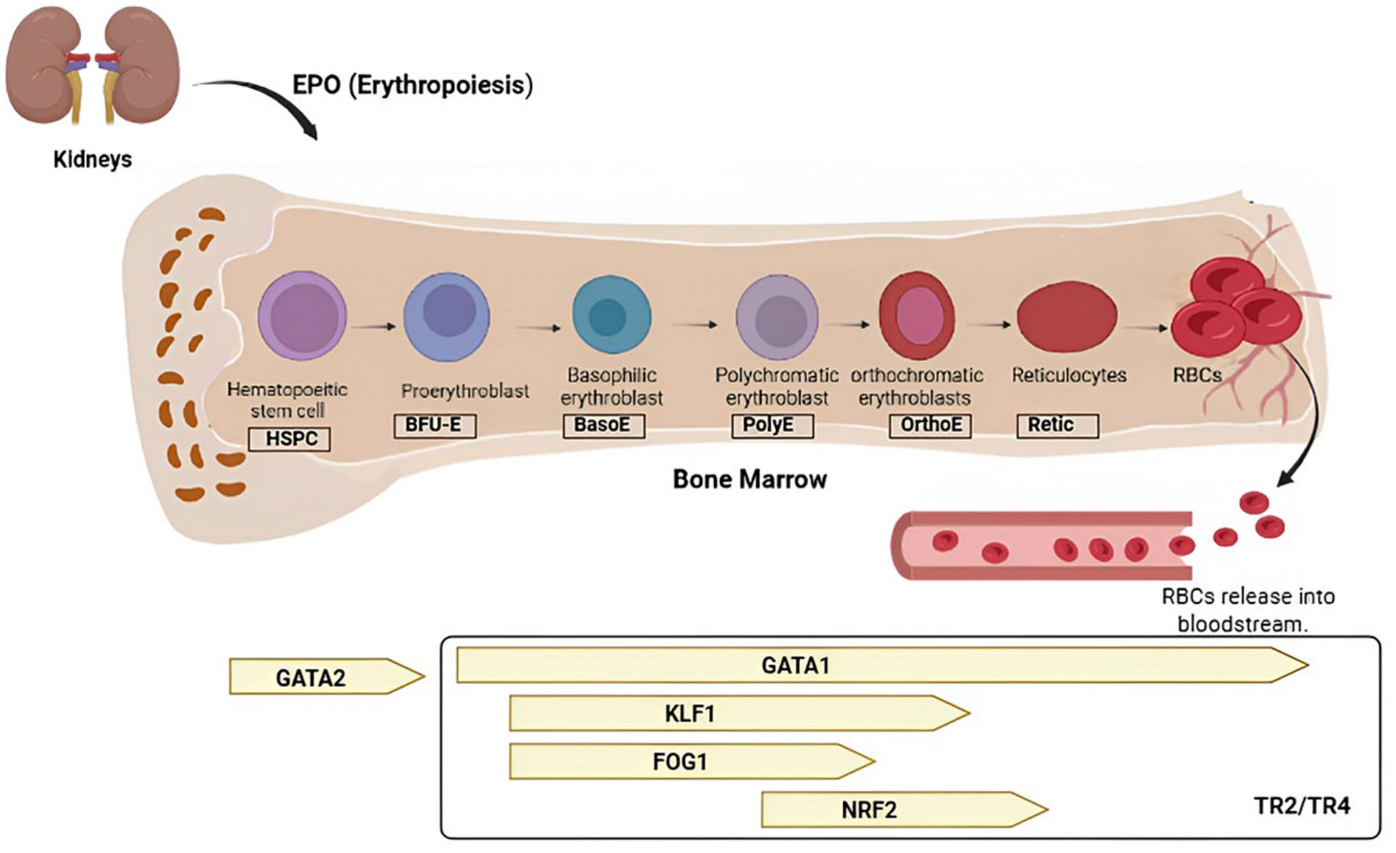
Erythropoiesis initiates with hematopoietic stem cells in the bone marrow and advances through sequential stages of erythroblast maturation (proerythroblast → basophilic erythroblast → polychromatic erythroblast → orthochromatic erythroblast) prior to enucleation, resulting in reticulocytes that develop into circulating red blood cells (RBCs). This process is stringently regulated by erythropoietin (EPO), a cytokine produced by the kidneys that activates JAK2/STAT5 signaling, increases anti-apoptotic BCL-xL expression, and facilitates hemoglobin synthesis and membrane protein assembly. A dynamic transcriptional network overlays this hormonal regulation: GATA2 promotes early progenitor proliferation, but its substitution by GATA1 triggers terminal erythroid differentiation by inducing globin genes, heme biosynthesis enzymes, and survival pathways. FOG1, functioning as a cofactor of GATA1, regulates its transcriptional activity, while KLF1 (EKLF) is essential for β-globin expression and the development of membrane protein systems vital for red blood cell integrity. Simultaneously, NRF2 protects erythroid precursors from oxidative stress during hemoglobin synthesis, whereas the nuclear receptors TR2/TR4 inhibit embryonic globin expression to facilitate the developmental shift to fetal and adult hemoglobins. The coordinated action of these pathways guarantees the accurate timing, reliability, and robustness of erythropoiesis, ultimately allowing RBCs to fulfill their particular role in efficient oxygen transport. Created with BioRender.com.

Erythropoiesis, the process of RBC maturation, originates in the bone marrow from multipotent hematopoietic stem cells that undergo lineage commitment under the influence of hematopoietic growth factors, with erythropoietin (EPO) being the most critical regulator (10, 12, 13). EPO is principally synthesized in the kidneys, and in response to hypoxic stimuli, it triggers the differentiation of progenitor cells into erythroblasts (14). These erythroblasts subsequently undergo sequential morphological and biochemical transformations, ultimately reaching a stage that is phenotypically similar to, yet distinct from, reticulocytes (10). The enucleation of erythroblasts represents a pivotal step in this maturation cascade, facilitating maximal hemoglobin accumulation while ensuring minimal metabolic activity (15, 16). Reticulocytes, which retain residual ribosomes and mitochondria, are then released into the circulation, where they complete their terminal maturation by losing organelles to form fully functional erythrocytes (3, 20). The remodeling of lipid and protein constituents within the erythrocyte membrane encompasses cytoskeletal composition, membrane-skeleton organization, and the anchoring and spatial arrangement of membrane proteins. These coordinated processes enable the acquisition of the characteristic biconcave discoid morphology and provide the mechanical resilience essential for optimal red blood cell function (1, 4). Molecular aspects of erythropoiesis involve a number of transcription factors that are involved in the regulation of erythroid-specific genes, such as GATA1, FOG1, and EKLF (19). These factors govern hemoglobin production, membrane protein assembly, and the enucleation process, enabling the transition of red blood cells from nucleated precursors to mature, enucleated cells specialized for efficient oxygen transport (19) (Figure 2). In hypoxic settings, erythropoiesis is meticulously regulated by the activation of the hypoxia-inducible factor (HIF) pathway, which largely enhances erythropoietin (EPO) synthesis in renal peritubular cells (53). EPO serves as a primary hormonal regulator that enhances the survival, proliferation, and differentiation of erythroid progenitors through the activation of JAK2/STAT5 signaling pathways. In addition to its traditional function in erythropoiesis, recent findings suggest that EPO signaling also affects red blood cell metabolism and membrane composition via regulating oxidative stress responses, cytoskeletal architecture, and lipid remodeling (54).

In pathological circumstances like chronic kidney disease (CKD), diminished EPO production resulting from renal dysfunction disturbs this regulatory axis, causing inefficient erythropoiesis and anemia. Besides diminished RBC production, CKD is linked to metabolic and structural changes in circulating erythrocytes, such as heightened oxidative stress, compromised membrane protein integrity, and decreased deformability. Moreover, hypoxia-induced signaling pathways facilitate adaptive modifications in erythrocyte metabolism, encompassing changes in glycolytic flux and levels of 2,3-bisphosphoglycerate (2,3-BPG), which influence oxygen delivery efficacy. The HIF–EPO axis serves as a crucial connection between environmental oxygen levels and red blood cell molecular homeostasis, with important consequences for physiological adaptation and disease development (55).

RBCs are short-lived cells in the human body that circulate for a lifespan of 100–120 days before undergoing senescence (15, 21). It has been reported that oxidation and other cumulative age-related changes cause a gradual decrease in the fluidity of the membrane, and the constant rigidity of the cytoskeleton makes senesced RBCs firm (21). With time, the dynamic properties of spectral protein and actin interaction decrease, which translate to decreased deformability. Consequently, senescent RBCs are less efficient at traversing narrow capillaries, particularly in the spleen, where they must pass through the splenic cords for functional testing (22).

A distinct biochemical component also contributes significantly to RBC senescence in parallel with mechanical degradation. One of the hallmark features of RBC senescence is the externalization of phosphatidylserine (PS), which serves as an “eat-me” signal for macrophage-mediated clearance. This process reflects underlying disruptions in membrane asymmetry and energy-dependent regulatory mechanisms (15, 23, 24).

Together with opsonins, these antibodies facilitate the recognition of senescent RBCs by phagocytic cells, leading to their clearance predominantly in the spleen and liver as part of the reticuloendothelial system. This process of erythrophagocytosis ensures the recycling of red blood cell components, particularly the iron derived from hemoglobin, for reuse within the body (15, 21).

The balance between RBC erythropoiesis and senescence is crucial for maintaining homeostasis in the bloodstream. Dysregulation in either process can lead to anemia or other blood disorders, highlighting the importance of understanding the molecular mechanisms involved in RBC maturation and aging (25, 54).

### RBCs efficiency in oxygen transport and molecular regulation

Oxygen transport by RBCs is orchestrated through precise molecular regulation, primarily dependent on the structural conformational transitions of hemoglobin and the influence of its molecular interactants. Hemoglobin, a globular protein with four subunits, two alpha and two beta chains, undergoes a conformational shift between its oxygenated (R) and deoxygenated (T) states, which is central to its function (35, 56). This shift is due to molecular processes and mechanisms that modulate the oxygen affinity of hemoglobin according to the physiological requirements of the various tissues (35, 56).

Cooperative binding is at the center of this regulation, whereby the binding of oxygen to one heme group makes the other heme groups in a better position to bind with oxygen (35). This allosteric mechanism ensures that again hemoglobin can quickly bind with oxygen in the high partial pressure area of the lungs. After binding of oxygen, the molecule switches from T, which has a low affinity for oxygen, to R state which has high oxygen affinity (56). This transition is due to sII opening and the distortion of intramolecular interactions causing the breaking of salt bridges and making the subsequent oxygen binding process less demanding (34, 35, 56). The reverse occurs in the peripheral tissues, where oxygen tension is lower. The opposite occurs in peripheral tissues, where the lower oxygen tension promotes the transition of hemoglobin to the T state, facilitating oxygen release to areas of greatest need. This process is regulated by several molecular factors that influence hemoglobin function (34, 35, 56).

**Figure 3:**
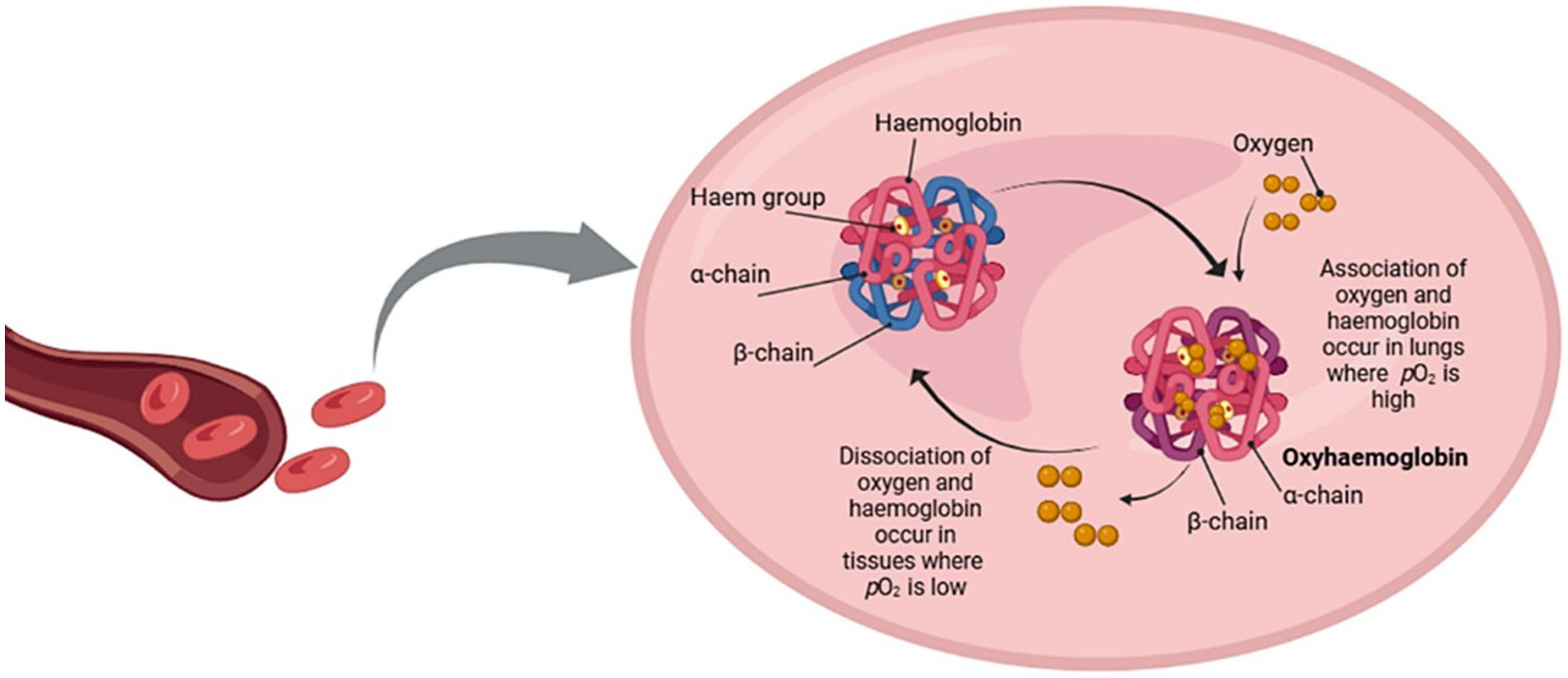
Hemoglobin is a tetrameric protein composed of two α-chains and two β-chains, each containing a heme group capable of binding oxygen. In the lungs, where the partial pressure of oxygen (pO_2_) is elevated, oxygen binds to hemoglobin to create oxyhemoglobin. In peripheral tissues with low partial pressure of oxygen (pO_2_), oxygen dissociates from hemoglobin, facilitating its release to sustain cellular metabolism. This reversible binding is meticulously controlled by allosteric effectors, such as 2,3-bisphosphoglycerate (2,3-BPG), carbon dioxide (CO_2_), and hydrogen ions (H^+^), which adjust hemoglobin’s oxygen affinity and promote effective gas exchange under diverse physiological situations. Created with BioRender.com.

One of the key regulators of hemoglobin’s affinity for oxygen is 2,3-bisphosphoglycerate (2,3-BPG), a metabolite produced in RBCs during glycolysis (32). 2,3-BPG binds to the central cavity of the deoxygenated form of hemoglobin, stabilizing the T state and thus reducing its affinity for oxygen (32). This binding is essential in ensuring that hemoglobin releases oxygen in tissues with lower oxygen pressure. Under conditions such as hypoxia or high altitude, the concentration of 2,3-BPG rises, enhancing oxygen unloading to compensate for diminished oxygen availability (57). This adaptive mechanism highlights the fine molecular control over oxygen delivery based on environmental and physiological conditions (32) (Figure 3).

Hemoglobin’s affinity for oxygen is also influenced by the Bohr effect, a crucial molecular mechanism regulating oxygen binding and release (56). The Bohr effect describes how increased concentrations of protons (H^+^) and carbon dioxide (CO₂) in the tissues lead to a decreased affinity of hemoglobin for oxygen. CO₂ produced as a metabolic byproduct, diffuses into RBCs where it is rapidly converted by the enzyme carbonic anhydrase into bicarbonate (HCO₃^−^) and protons. These protons bind to specific residues on hemoglobin, stabilizing the T state and promoting oxygen release (58). This interaction is essential for oxygen delivery to metabolically active tissues, which typically generate more CO₂ and acid, thus creating conditions that favor oxygen unloading. Conversely, in the lungs, where CO₂ is expelled, and pH rises, the affinity of hemoglobin for oxygen increases, allowing efficient oxygen binding.

Nitric oxide (NO) interaction with hemoglobin represents an additional molecular mechanism regulating oxygen transport (56). NO, a potent vasodilator produced by endothelial cells, plays a critical role in modulating blood flow and, consequently, oxygen delivery. Hemoglobin can bind NO, acting as a carrier that influences vascular tone. In hypoxic conditions, NO is released, leading to vasodilation, which increases blood flow to oxygen-deprived tissues (56). This interaction ensures that oxygen delivery is tightly coupled with the metabolic demands of tissues, further enhancing the efficiency of gas transport.

At the structural level, the deformability of RBCs is controlled by the cytoskeleton which is critical for oxygen transport. The spectrin-actin network offers the mechanical pliability required for RBCs to distort while passing through slim blood vessels (6). By clearly detailing that force and energy transmission between cytoskeletal proteins and the membrane depends on band 3 and ankyrin, it is possible to conclude that the shape of RBCs remains biconcave (6, 8). This shape also provides a broad surface area for gas exchange while equipping RBCs to withstand mechanical stress without rupturing (6, 37). As RBCs age, oxidative damage accumulates, weakening the cytoskeletal network and reducing cell flexibility, which impairs oxygen delivery (59). The structural integrity of the cytoskeleton is therefore instrumental in defining RBC function, and shows how molecular structure relates to physiological function (6, 36).

The chloride shift mechanism enables RBCs to facilitate the elimination of CO₂ from tissues. After carbonic anhydrase catalyzes the formation of bicarbonate from CO₂, bicarbonate is transported out of the RBC in exchange for chloride ions (Cl^−^) (60). This exchange, mediated by band 3, ensures that CO₂ is efficiently carried in the form of bicarbonate, which is more soluble in plasma. In the lungs, the reverse reaction occurs, allowing CO₂ to be exhaled and oxygen to bind hemoglobin for delivery to tissues (60). This tightly regulated molecular process ensures that gas exchange is continuous and efficient, adapting to the varying demands of the body.

The molecular regulation of RBC function encompasses a complex interplay of hemoglobin’s allosteric properties, its interactions with small molecules like 2,3-BPG and nitric oxide, and the structural integrity of the cytoskeleton (32). These mechanisms safeguard the dynamic response of the RBCs to changes in oxygen and CO₂ levels, allowing them to efficiently transport oxygen and remove CO₂ throughout a range of physiological conditions (32, 60). This molecular sophistication highlights the critical role of the RBC in maintaining homeostasis and fulfilling the metabolic requirements of tissues.

## Factors affecting RBCs function

The functioning and viability of RBCs are affected by numerous intrinsic and extrinsic variables that collectively limit their capacity to facilitate oxygen transport and maintain homeostasis in circulation. Aging-related molecular changes are among the most critical factors influencing RBC function. As RBCs age, they gradually acquire biochemical and structural alterations that compromise membrane integrity, deformability, and metabolic function. These modifications not only hinder their microcirculatory movement but also function as molecular signals that facilitate their identification and removal by the reticuloendothelial system.

### Age-related molecular changes in RBCs

Aging RBCs undergo progressive molecular changes that compromise their structure and function. These include reduced membrane fluidity, loss of surface sialic acid, increased oxidation of membrane proteins, alterations in phospholipid asymmetry, diminished enzymatic activity, and the accumulation of damaged proteins (15, 21–23, 36, 55, 61). In addition, aged red blood cells show increased binding of immunoglobulin G (IgG) (15, 21). Together, these modifications reduce cellular deformability and ultimately signal the spleen to remove senescent red blood cells from circulation (15, 21, 22, 61).

Age-dependent alterations in membrane fluidity and protein composition significantly affect RBC efficiency and viability. Over time, membrane deformability declines due to changes in lipid content and oxidative damage to proteins (61, 62). These modifications impair the cell’s flexibility, hindering its ability to traverse narrow capillaries (15, 21, 61). Furthermore, the loss of sialic acid from the cell surface reduces membrane stability and diminishes the negative surface charge that normally prevents red blood cell aggregation (1, 2, 22).

RBCs are continuously exposed to oxidative stress throughout their lifespan due to the generation of reactive oxygen species (ROS). This results in the formation of methemoglobin and cross-linking of membrane proteins, which increase susceptibility to further oxidation and reduce the efficiency of gas transport (55). Oxidative modifications of cytoskeletal proteins, including but not limited to spectrin, disrupt the filamentous network and diminish membrane elasticity (6, 9, 36, 55). In addition, oxidative stress alters the organization of membrane phospholipids, a key determinant of red blood cell vitality, stability, and functional efficiency (23, 36, 45, 55).

ROS are essential for promoting oxidative damage in red blood cells, especially via lipid peroxidation of the membrane bilayer (36). Red blood cells have been particularly susceptible to oxidative stress due to their constant exposure to oxygen and elevated iron levels. ROS engage with polyunsaturated fatty acids in membrane phospholipids, triggering lipid peroxidation pathways that produce reactive aldehydes, including malondialdehyde (MDA) and 4-hydroxynonenal (4-HNE). These byproducts exacerbate oxidative damage by creating cross-links with membrane proteins and cytoskeletal elements (23, 36). Lipid peroxidation affects membrane fluidity and weakens the structural integrity of the lipid bilayer, resulting in enhanced membrane stiffness and diminished deformability (36). This impairment restricts the capacity of RBCs to navigate the microvasculature effectively, especially in the splenic circulation (23). Furthermore, oxidative alteration of membrane lipids leads to the disruption of phospholipid asymmetry, promoting the externalization of phosphatidylserine, which serves as a pro-clearance signal for macrophage identification (24). Moreover, ROS-induced lipid damage promotes membrane vesiculation and expedites erythrocyte senescence, hence reducing RBC lifespan (23, 36, 63). These mechanisms are exacerbated in pathological diseases such as hemoglobinopathies, metabolic disorders, and chronic kidney disease, characterized by heightened oxidative stress (55). Lipid peroxidation serves as a pivotal biochemical pathway connecting oxidative stress to diminished RBC deformability, longevity, and clearance, significantly influencing disease progression and transfusion results (23, 36, 55).

RBC senescence is marked by specific molecular alterations that signal their clearance from circulation. Among these, the externalization of PS on the outer membrane leaflet and the accumulation of band 3-ankyrin complexes are recognized hallmarks of aged erythrocytes (18). PS is normally located in the inner leaflet and is anchored by means of ATP-dependent flippases under physiological conditions. During the senescence of RBCs and where energy yield is low, PS is externalized to act as an “eat me” signal to be engulfed by macrophages. Other highlights are the unmasking of the band 3 proteins, which is involved in the gathering of naturally occurring antibodies and opsonins. These molecular signals enable the recognition by macrophages in the spleen and liver and are well described by the ELM 3 module. The old RBCs are removed through phagocytosis and recycled their components for reuse, such as iron (18). This tightly regulated process of erythrophagocytosis ensures the renewal of functional circulating red blood cells, thereby maintaining hemostasis and preventing the accumulation of abnormal erythrocyte forms.

### Geographic differences in RBC molecular composition

The molecular structure of RBCs exhibits significant variability among human cultures, indicative of evolutionary influences shaped by geographic and environmental factors. This is particularly evident in high-altitude areas, where prolonged hypobaric hypoxia requires essential modifications in oxygen delivery and cellular function. The hypoxia-inducible factor (HIF) signaling cascade is important to these adaptations, serving as a principal regulator of oxygen detection that coordinates transcriptional programs affecting erythropoiesis, angiogenesis, and intermediate metabolism. Variants in EPAS1 and EGLN1 proteins, prevalent among Tibetan highlanders, illustrate population-specific genetic fingerprints that modulate the erythropoietic response to prolonged hypoxia and inhibit maladaptive erythrocytosis (64, 65).

The hematological phenotypes resulting from these molecular bases vary significantly among highland populations. Andean populations demonstrate a typical polycythemic response marked by heightened hemoglobin levels and increased erythrocyte mass, a mechanism that enhances arterial oxygen content but imposes the pathophysiological burden of elevated blood viscosity and susceptibility to chronic mountain sickness (CMS) (65). Tibetans sustain hemoglobin levels similar to those at sea level while ensuring efficient oxygen delivery through precise regulatory mechanisms, such as modified hemoglobin isoform dynamics and regulation of HIF-responsive genes. Such modifications mitigate the risk of CMS and illustrate an alternate evolutionary pathway for maintaining tissue oxygenation (64, 66).

The Ethiopian highlanders exhibit a distinct paradigm. The Amhara population maintains stable hemoglobin levels alongside increased NO bioavailability, promoting vasodilation and effective microvascular perfusion, while the Oromo population display a phenotype resembling the Andean model, where hemoglobin elevation serves as the primary compensatory mechanism (67). In addition to hemoglobin concentration and isoform variety, hypoxia triggers the modification of RBC membrane proteins and ion transport mechanisms. The upregulation of the Gardos channel facilitates ionic fluxes that govern cell hydration and volume, while conformational alterations of Band 3 and spectrin improve cytoskeletal plasticity, allowing RBCs to maintain deformability under heightened shear stress and microvascular resistance (65). These molecular modifications alleviate the rheological difficulties of circulation in hypoxic conditions and diminish the probability of premature erythrocyte clearance.

Collectively, our comparative findings highlight that the adaptive landscape of high-altitude populations is neither uniform nor gradual but rather consists of discrete molecular strategies influenced by unique evolutionary histories. Andean erythrocytosis, Tibetan genetic control of HIF signaling, and Ethiopian endothelial-vascular adaptations exemplify convergent responses to the identical physiological challenge of oxygen deficiency, each realized through distinct molecular routes. This variation underscores the adaptability of RBC molecular biology under selective pressure and emphasizes the need for population-specific frameworks for examining the pathophysiology of hypoxia and its translational implications in hematology.

### Disease-related molecular differences in RBCs

RBCs undergo significant alterations in molecular composition, morphology, and lifespan under various disease states. Depending on the underlying condition, these alterations can be broadly categorized into hematologic disorders, metabolic disorders, and infectious diseases (61).

#### Hematological disorders

Sickle cell disease is caused by a single point mutation of the β-globin gene, which leads to the synthesis of abnormal hemoglobin S (HbS). In the absence of oxygen, HbS undergoes polymerization, and RBCs appear morphologically sickle-shaped (68). This structural change interferes with membrane proteins such as ankyrin and spectrin and enhances the stiffness and the frailty of the cells (6, 8, 9, 36). Further, by creating an excessively oxidative environment, oxidative stress causes an increased level of hemolysis, resulting in chronic anemia (23, 36, 55). These dysfunctional RBCs make the path to microvasculature equivalently torturous as they lead to ischemia and pain crises (11, 15, 52).

Thalassemia can be defined as a genetic disorder which, under the circumstances of α- or β-globin chain anomalies, presents with impaired or completely deficient synthesis of the hemoglobin (69, 70). This results in an unequally shared hemoglobin anomalous proclivity, which results in oxidative stress and aberrations in membrane stability. This is because the lifespan of RBCs is reduced due to early loss of the spleen in patients with thalassemia. Microcytic and hypochromic cells, along with the presence of membrane instability already decrease the count and efficiency of an RBC to deliver oxygen, thus explaining clinical manifestations of the disease (33, 70).

In hemoglobinopathies like β-thalassemia, inefficient erythropoiesis serves as a primary pathogenic mechanism that significantly influences RBC maturation and molecular phenotype. This process results from disproportionate globin chain synthesis, where excess unpaired α-globin chains aggregate inside erythroid precursors, causing oxidative damage, membrane instability, and the initiation of apoptotic pathways. A substantial percentage of erythroid precursors experience premature demise in the bone marrow prior to achieving full maturity (33, 69).

This disruption of erythropoiesis diminishes effective RBC production and results in the release of structurally abnormal erythrocytes into circulation (69). These cells display modified membrane protein composition, encompassing changes in spectrin, ankyrin, and band 3 interactions, alongside increased oxidative damage and phospholipid asymmetry. As a result, circulating red blood cells exhibit diminished deformability, higher susceptibility to hemolysis, and a decreased lifespan. Ineffective erythropoiesis also leads to systemic problems due to prolonged hypoxia, iron overload, and the growth of the erythroid compartment, which intensifies disease severity (57). Ineffective erythropoiesis creates a clear mechanistic connection between genetic abnormalities in hemoglobin production and the modified molecular and functional characteristics of circulating red blood cells, underscoring its essential involvement in disease pathogenesis and therapeutic intervention (33).

#### Metabolic diseases

Metabolic disorders affecting red blood cells encompass a wide range of enzymatic and biochemical abnormalities that disrupt cellular homeostasis and survival. In diabetes, raised glucose levels for a long time cause the glycation of the RBC membrane, spectrin, and band 3 proteins. This glycation leads to changes in membrane flexibility and reduced flexibility of the RBC in relation to providing passage through micro capillaries. Glycated RBCs are also more sensitive to oxidative stress, which injures the lipid bilayer and shortens the life span of the cell. By altering the features of blood flow, these actions beget complications of the disease, including microvascular damage and tissue hypoxia (36, 52).

Glucose-6-phosphate dehydrogenase (G6PD) insufficiency is a highly widespread enzymopathy impacting red blood cells globally and illustrates disrupted redox metabolism. G6PD is pivotal in the pentose phosphate pathway, producing NADPH, which is crucial for sustaining decreased glutathione levels and safeguarding erythrocytes from oxidative damage. A deficiency of G6PD undermines the antioxidant defense system, heightening vulnerability to oxidative stress, hemoglobin denaturation, and membrane instability, which finally culminates in hemolysis (71, 72).

G6PD deficiency is clinically correlated with regional distribution patterns, especially in malaria-endemic areas, where it provides partial protection against severe malaria. Nonetheless, exposure to oxidative stresses, such as infections, some medicines, and fava beans, can trigger acute hemolytic episodes.

Glucose-6-phosphate dehydrogenase (G6PD) deficiency has important implications in transfusion medicine, particularly in relation to RBC storage and post-transfusion efficacy. G6PD plays a critical role in maintaining intracellular redox balance through NADPH generation; therefore, deficient RBCs exhibit impaired antioxidant capacity and increased susceptibility to oxidative stress. During storage, these cells are more prone to oxidative damage, including lipid peroxidation, protein oxidation, and membrane instability, which collectively contribute to accelerated storage lesion development (73–75).

As a result, transfusion of G6PD-deficient RBCs may be associated with reduced post-transfusion survival, increased hemolysis, and compromised oxygen delivery, particularly in recipients exposed to oxidative stress conditions such as infection or inflammation (71, 76). These findings highlight the importance of considering donor enzymatic status in transfusion practice and underscore the need for further research into screening strategies and storage optimization for G6PD-deficient units. Integrating enzymopathies into transfusion-related discussions strengthens the metabolic and translational relevance of RBC research and provides a more comprehensive understanding of factors influencing transfusion outcomes.

Chronic kidney disease (CKD) is characterized by reduced RBC production and introduces crucial changes in the structure and function of those cells. According to the study uremic toxins induce oxidative stress and affect the compositions of the membrane, affecting proteins such as ankyrin and band 3 (55). CKD also diminishes RBC deformability, limiting their ability to adapt to capillary flow. Such changes, along with erythropoietin deficiency, accelerate anemia and minimize overall oxygen transport capacity in patients with CKD (55).

#### Infectious diseases

Malaria, especially that caused by *Plasmodium falciparum* parasite, is known to exert a molecular impact on RBC to the extreme. This parasite enters RBCs and rearranges their membrane by translocating proteins like PfEMP1 to the outermost cell membrane (31). These proteins induce attachment to endothelial cells and are not cleared by the spleen, leading to microvascular occlusion and major clinical events. In addition, the parasite induces modifications in ion channels, thereby increasing membrane permeability and enhancing nutrient uptake essential for its survival. These alterations promote oxidative stress and compromise membrane integrity, ultimately leading to hemolysis and anemia (25, 31).

Infected HIV indirectly alters RBC molecular composition through chronic inflammation and oxidative stress. Raising ROS levels leads to the injury of membranes and proteins, which causes uncompensated loss of cholesterol and existential modification, contributing to RBC’s instability and deformability problems. In addition, the antiretroviral therapy, which is used to treat HIV, creates additional oxidative stress. The combined result tends to involve a reduction in the RBCs’ lifespan and an increased susceptibility to anemia, which is a frequent manifestation in people with HIV (31).

These studies collectively highlight that RBCs are active participants in the pathophysiology of a range of illnesses. Findings show that infections, genetic abnormalities, and chronic metabolic diseases intersect at common pathways: oxidative stress, membrane remodeling, and reduced deformability. This suggests that RBCs function as both targets and transmitters of systemic pathology. Overall, current evidence indicates that RBC molecular changes may play a role in disease progression rather than being solely secondary effects.

## Comparative molecular mechanisms in environmental, pathological, and physiological conditions

RBCs exposed to physiological aging, pathological disorders, and environmental stressors exhibit a limited set of converging molecular alterations that directly impact membrane integrity, deformability, and survival. A key factor in all of these situations is oxidative stress, which encourages lipid peroxidation, band 3 clustering, and phospholipid asymmetry disruption, all of which lead to early clearance (23, 36). Concurrently, altered cell hydration, increased membrane stiffness, and decreased microcirculatory efficiency are caused by disruptions in ion homeostasis, especially through PIEZO1 and Gardos channel activation (57).

Despite these common pathways, the initial mechanisms and clinical consequences diverge. Hemoglobinopathies are defined by an imbalance of globin chains and inadequate erythropoiesis, resulting in premature oxidative damage and the release of structurally impaired red blood cells with a reduced lifespan (33, 69). Aging signifies a cumulative deterioration in oxidative and metabolic functions, characterized by diminished ATP-dependent membrane maintenance and an incremental loss of deformability (23). Hypoxic and environmental conditions trigger adaptive responses through hypoxia-inducible factor (HIF) signaling and erythropoietin (EPO), leading to metabolic regulation and changes in oxygen delivery capacity (44, 54).

From a transfusion standpoint, these discrepancies are notably significant since similar biological processes may yield divergent functional consequences dependent upon the underlying circumstances. Oxidative damage and membrane remodeling contribute to *in vivo* red blood cell senescence and storage-related defects, impacting post-transfusion recovery and efficacy. These findings support a membrane-centered model of red blood cell regulation, in which structural, metabolic, and oxidative changes are integrated at the membrane level and collectively determine erythrocyte function, aging, and clearance (77). Table 1 presents a comparative review of these mechanisms, emphasizing both common pathways and condition-specific distinctions.

**Table 1:** Comparative molecular alterations in red blood cells across physiological aging, pathological conditions, and environmental stressors.

Feature	Physiological aging	Pathological conditions (e.g., hemoglobinopathies)	Environmental/hypoxic conditions
Oxidative stress	Gradual accumulation of ROS leading to mild lipid and protein oxidation (23)	Marked oxidative stress due to globin imbalance and iron overload (33, 36)	Increased ROS with adaptive antioxidant responses (36, 44)
Membrane integrity	Progressive loss of membrane flexibility and protein stability (23)	Significant membrane damage with cytoskeletal disruption (33, 69)	Adaptive remodeling with variable membrane stability (44)
Ion homeostasis	Mild impairment in ion transport and ATP-dependent pumps (23)	Dysregulated ion channels (PIEZO1, Gardos) causing dehydration and rigidity (57)	Adaptive ion transport changes to maintain oxygen delivery (44, 57)
Phospholipid asymmetry (PS exposure)	Increased PS externalization as a senescence signal (23)	Enhanced PS exposure promoting premature clearance (69)	Mild or transient changes depending on hypoxic stress (44)
Deformability	Gradual reduction due to membrane and cytoskeletal aging (23)	Markedly reduced due to oxidative damage and membrane instability (33, 57)	Variable; may be preserved or transiently altered (44)
Erythropoiesis	Normal turnover with balanced production and clearance (23)	Ineffective erythropoiesis with precursor destruction (33, 69)	Increased erythropoiesis via HIF–EPO signaling (44, 54)
Metabolic state (ATP, glycolysis)	Decline in ATP levels with aging (23)	Altered metabolism with increased oxidative burden (57)	Adaptive metabolic shifts (↑ glycolysis, ↑ 2,3-BPG) (44, 54)

## Implications for clinical practice and future research

An enhanced comprehension of the molecular changes that regulate RBC function has reshaped clinical diagnostics and treatment approaches. Molecular biology establishes a framework for precision medicine in hematology and transfusion practice by correlating protein and lipid abnormalities with inherited and acquired illnesses (77). The identification of molecular markers and regulatory pathways enhances diagnostic precision and facilitates tailored therapies that reduce hemolysis, improve transfusion results, and elevate long-term patient care (78). These processes are also central to the development of storage lesions, where biochemical and structural changes during storage impair post-transfusion recovery and function (73, 74).

Diagnostic applications have most prominently benefited from advancements in molecular characterization. Proteins including Band 3, glycophorins, CD47, and mechanosensitive ion channels such as PIEZO1 serve as crucial facilitators of RBC activity and as sensitive markers of pathological alterations. Genetic screening for PIEZO1 mutations facilitates the early identification of hereditary xerocytosis, whereas the assessment of Gardos channel function provides diagnostic precision in instances of RBC dehydration and sickling disorders (68). In addition to genetics, membrane protein and lipid profiling using flow cytometry and mass spectrometry offers a functional assessment of RBC health. The externalization of phosphatidylserine on the outer membrane leaflet acts as a measurable biomarker for apoptosis and hemolytichemolytic anemia (31). Likewise, the overexpression of adhesion molecules like ICAM-4 and CD36 is associated with the severity of vascular problems in sickle cell aneamia and malaria, and their monitoring may offer clinicians important metrics for evaluating disease progression and treatment effectiveness. These diagnostic methods collectively establish molecular markers as essential tools for guiding prognosis and personalized therapeutic planning (51, 79).

Therapeutic approaches guided by RBC molecular biology are progressively influencing the treatment of hematological disorders. Ion channel modulators, specifically those aimed at PIEZO1 and the Gardos channel, provide a method to restore ionic equilibrium, diminish dehydration, and mitigate hemolysis in conditions like hereditary xerocytosis and sickle cell disease (80). The stabilization of Band 3 protein integrity is a viable approach to mitigate oxidative damage and maintain RBC flexibility. Interventions that improve phospholipid asymmetry by modulating flippase or scramblase activity potentially aided by antioxidants like ascorbate may inhibit premature phosphatidylserine exposure, postpone pathological clearance, and mitigate vascular obstruction in thalassemia and sickle cell anemia (80).

A critical translational challenge is the enhancement of RBC preservation conditions. In refrigerated storage, erythrocytes develop “storage lesions,” characterized by oxidative alterations of membrane lipids, fragmentation of essential cytoskeletal proteins, and a gradual decline in deformability. These alterations diminish post-transfusion longevity and may lead to negative clinical consequences. Therapeutic addition with antioxidants, ion channel modulators, or metabolic enhancers during storage may prolong the shelf-life and functionality of preserved RBC units, hence augmenting transfusion safety (73). The manipulation of adhesion molecules, including CD47, ICAM-4, and CD36, provides innovative therapeutic opportunities. Modulating CD47–SIRPα signaling may regulate RBC clearance in autoimmune hemolytic anemia, whereas attenuating ICAM-4 and CD36 activity could mitigate vascular occlusion and inflammatory adhesion in sickle cell disease (48).

Progress in molecular hematology is facilitating the creation of focused treatment approaches designed to rectify specific red blood cell anomalies at the molecular level. The modulation of ion channels, specifically PIEZO1 and the Gardos channel, offers a promising strategy to restore ionic equilibrium, mitigate cellular dehydration, and enhance deformability in conditions such as sickle cell disease and hereditary xerocytosis (17, 35). Targeting oxidative stress pathways using antioxidant or redox-modulating therapy may enhance membrane integrity, reduce lipid peroxidation, and prolong erythrocyte longevity (36).

Novel methodologies emphasize the stabilization of membrane–cytoskeleton contacts and the preservation of phospholipid asymmetry to prevent premature clearance. The incorporation of molecular diagnostics, such as proteomic and lipidomic profiling, presents novel prospects for individualized risk assessment and treatment enhancement (55, 57). In transfusion medicine, these discoveries contribute to advancements in storage methodologies, including metabolic rejuvenation and focused modulation of membrane pathways to mitigate storage lesions and enhance post-transfusion effectiveness (80). The combination of molecular profiling, precision medicine, and targeted medicines is anticipated to revolutionize the management of hematological illnesses, facilitating personalized treatment approaches and enhancing clinical results and transfusion safety (57, 80).

In summary, progress in molecular hematology has placed RBC proteins and lipids at the forefront of clinical innovation. Diagnostic assays utilizing these markers now deliver exceptional sensitivity in disease detection and monitoring, while therapeutic interventions aimed at the same pathways present customized strategies to alleviate RBC fragility, enhance transfusion results, and diminish the impact of inherited and acquired anemias. These advancements underscore that the molecular lens elucidates the pathogenesis of RBC diseases while enhancing their clinical management, establishing the stability and functionality of RBCs as the definitive criterion for therapeutic success.

## Conclusion

RBCs have been defined as the key organs for oxygen carriage and regulation of endogenous/exogenous balance. Such information is important for managing a variety of diseases affecting the hematologic system, as well as for the elucidation of diagnostic precision and the advancement of medical treatments. In this review, critically important molecules, including membrane proteins, ion channels, phospholipids, glycoproteins, and adhesion molecules involved in the physiological process of RBC and their pathophysiology, have been discussed. These molecules regulate RBC deformability, survival, and behavior in vascular compartments and are critical to disease and health. New molecular markers consisting of band 3, PIEZO1, and glycophorins have a favorable result for diagnostics. These genetic changes in these molecules are the basis for the study of diseases’ causative factors by establishing factors such as early diagnosis and improved profiles on managing conditions like hereditary xerocytosis, sickle cell anemia, and autoimmune hemolytic anemia. Thus, the capacity to detect alterations in RBC molecular signatures increases disease surveillance and therapy assessment, which leads to better patient prognosis.

Pharmacological intervention and therapeutic strategies aimed to modify brain RBC molecular signatures have been identified and are greatly promising. Such phenomena as changing ion transportability, maintaining the structural integrity of cell membrane, and reducing pathologic adhesion molecule function could help to reduce treatment-related complications in a wide spectrum of diseases. Moreover, knowledge of molecular changes in RBC during storage has novel importance in transfusion medicine. Conventional crossmatched RBCs receive “storage lesions,” such as protein denaturation, lipid peroxidation, and decreased pliability, which affects their viability. Revolutions in handling and storage measures and procedures or medicines like antioxidant precursors or ion channel stabilizers make RBC survival longer and transfusions safer.

In conclusion, it is warranted that defining molecular mechanisms will provide better diagnostic information, therapeutic strategies, and prognostic factors in the disease course of RBC-associated disorders, thereby improving the quality of patients’ lives and the practice of transfusion medicine. Hence, the Identification of RBC molecular mechanisms assists in the development of diagnostic tests, therapies, and transfusions. Further investigation of these aspects of RBC biology shall improve not only our knowledge of MLVs but they shall improve the general well-being of society under conditions concerning the management of hematological diseases and blood storage for transfusion needs.
